# Evidence of a role for interleukin-6 in *anoikis* resistance in oral squamous cell carcinoma

**DOI:** 10.1007/s12032-022-01664-5

**Published:** 2022-04-29

**Authors:** Marilena Karavyraki, Richard K. Porter

**Affiliations:** School of Biochemistry and Immunology, Trinity College Dublin, Trinity Biomedical Science Institute (TBSI), Pearse Street, Dublin, D02 R590 Ireland

**Keywords:** SCC-4, *Anoikis* resistance, Metastasis, IL-6, TLR2, Pam2CSK4

## Abstract

In an endeavour to understand metastasis from oral squamous cell carcinomas, we characterised the metastatic potential of a human tongue derived cell line (SCC-4 cells) and compared this phenotype to pre-cancerous dysplastic oral keratinocyte (DOK) cells derived from human tongue and primary gingival keratinocytes (PGK). We demonstrate that SCC-4 cells constitutively synthesize and release significant amounts of IL-6, a process that is enhanced by the addition of the TLR2/TLR6 agonist, Pam2CSK4. The expression of TLR2/6 and IL-6Ra/gp130 receptors was also confirmed in SCC-4 cells. Cancerous SCC-4 human tongue cells also have a classic EMT profile, unlike precancerous human tongue DOK cells. We also established that IL-6 is driving *anoikis* resistance in an autocrine fashion and that anti-IL-6 neutralising antibodies, anti-IL-6 receptor antibodies and anti-TLR2 receptor antibodies inhibit *anoikis* resistance in cancerous SCC-4 human tongue cells*.* The data suggest a promising role for anti-IL-6 receptor antibody and anti-TLR2 receptor antibody treatment for oral cancer.

**Supplementary Information:**

The online version contains supplementary material available at 10.1007/s12032-022-01664-5.

## Introduction

Oral squamous cell carcinomas (OSCC) are the sixth most common type of cancer in the world and accounts for more than 90% of oral malignancies [1]. OSCC are metastatic cancers and arise from several anatomic sites within the oral cavity but most commonly from the tongue. Chemokines and cytokines (mostly interleukins) have been found to play key roles in cancer metastasis by, promoting the detachment and survival (*anoikis* resistance) of cancer cells from the primary tumour sites, affecting the epithelial to mesenchymal transition (EMT), regulating cancer cell migration and stimulating proliferation [2–4]. Normal cells undego apoptosis when they lose adhesion to the extracellular matrix (ECM), a process termed as “*anoikis*”, from the Greek phrase “without home”. Metastatic cancer cells develop the ability to survive and grow detached from the extracellular matrix and are thus termed *anoikis* resistant [5]. Cytokines IL-6 and IL-8 have been demonstrated to synergistically regulate a range of characteristics of cancer metastasis which were found to cooperatively regulate cancer cell proliferation, migration and the seeding of circulating tumour cells in various cancer cell types [6–8]. In addition, autocrine synthesis of IL-6 has been observed in a wide range of malignant tumours, including OSCC [9–11]. Furthermore, there is evidence that autocrine IL-6 production is an important element in tumour metastasis and enhancement of cell migration and invasion in human chondrosarcoma and osteosarcoma [12–14]. It is also noteworthy that *anoikis* resistance observed in pancreatic cells, was further increased upon IL-6 treatment [15]. In general, tumour metastasis to distant organs is a crucial step in establishing the aggressive phenotype of human cancer and it usually results in mortality of cancer patients [16]. TLR2, a member of the trans-membrane toll-like receptors family, is expressed on the surface of many cell types, including various cancer cell types and it is activated by several reported ligands [17–19]. Recent observations by Dong et al. [20] indicated a correlation between upregulated TLR2 expression and increased cell proliferation, invasion and migration of colorectal cancer. Additionally, inhibition of TLR2 signalling was demonstrated to suppress colorectal cancer cell growth, revealing a potential key role of TLR2 in colorectal tumorigenesis [20]. In this study we investigated TLR2 ligand-dependent IL-6 release and IL-6-dependent *anoikis* resistance in OSCC.

## Materials and methods

### PGK cell line

Primary gingival keratinocyte (PGK; cat# PCS-200-014) cells are human (jaw) adult derived gingival epithelial-like normal primary adherent cells, with a rounded, cobblestone appearance. PGK cells were mycoplasma-free and grown in cell culture flasks of Dermal Cell Basal Medium (DCBM) supplemented with 0.4% Bovine Pituitary Extract, 0.5 ng/ml rh TGFα, 6 mM l-glutamine, 100 ng/ml hydrocortisone, 5 mg/ml recombinant human insulin, 1.0 mM epinephrine, 5 mg/ml ApoTransferrin and penicillin–streptomycin (50 U/ml and 50 μg/Ml) (Gibco). Cells were grown at 37 °C in humidified environment containing 95% O_2_ and 5% CO_2_. PGK cells were passaged depending on their levels of confluency (75–80%), while medium was refreshed every 2–3 days. PGK cells, DCBM and ApoTransferrin were purchased from American Type Culture Collection (Manassas, VA, USA).

### DOK and SCC-4 cell lines

Dysplastic oral keratinocyte (DOK) cells were originally isolated from a piece of dorsal tongue of a 57-year-old male. DOK cells [cat# ECACC 94122104] are characterized as Caucasian derived epithelial adherent tongue dysplastic cells. Squamous cell carcinoma (SCC-4) cells were originally established from the tongue of a 55-year-old male [SCC-4 cat# ECACC 89062002]. Mycoplasma-free cells were grown in cell culture flasks in Dulbecco’s Modified Eagle’s Medium GlutaMAX cell culture medium (Gibco) supplemented with 5 µg/ml hydrocortisone, 20% (v/v) Foetal Bovine Serum (FBS) and penicillin–streptomycin (50 U/ml and 50 μg/ml) (Gibco). Cells were grown at 37 °C in humidified environment containing 95% O_2_ and 5% CO_2_. DOK and SCC-4 cells were passaged at least twice weekly depending on their levels of confluency (75–80%) and were purchased from the European Centre of Authenticated Cell Culture. In terms of validity of comparability, all cells used in this study originated from the human buccal cavity and represent primary (PGK), pre-cancerous (DOK) and cancerous (SCC4) cells, with the latter two being from the same tissue (tongue).

### Viability assay

AlamarBlue^*®*^ (Invitrogen) was employed to quantitatively measure the viability of cells. Resazurin, the active cell-permeable ingredient of alamarBlue^*®*^, has a blue colour and after entering the cells, is reduced to resorufin, which then produces a red fluorescence. Viable cells are able to convert resazurin to resorufin giving a quantitative measure of their viability, while non-viable cells with a worse innate metabolic activity produce less resorufin. Plates were read on a Spectramax Gemini Plate Reader using SOFTmax Pro version 4.9 (Molecular Devices, Sunnyville, C.A, U.S.A.) at excitation and emission wavelengths of 544 nm and 590 nm, respectively. Experiments were conducted in triplicate and RFU values were expressed as mean ± SEM of the three experiments.

### Measurement of cytokine concentration by ELISA

Commercially available ELISA kits were used according to manufacturer’s instructions. Once sufficient colour was developed, 20 μl/well stop solution (2 N H_2_SO_4_) was added and OD values were obtained by measure absorbance at 450 nm using Spectramax Microplate Reader (Molecular Devices). Concentrations of cytokines were determined using the standard curve from each ELISA plate. Commercial Human ELISA kit information: IL-6, (Biolegend/MSC); IL-8 (Biolegend/MSC); TNF-α (Biolegend/MSC); IL-11 (R&D Systems).

### Protein determination using the bicinchoninic acid (BCA) assay

Quantification of protein concentrations in cell lysates was carried out using the Bicinchoninic Acid (BCA) Assay as described by Smith et al*.* [21].

### SDS-PAGE and immunoblotting

SDS-PAGE and immunoblotting were performed as previously described by Geoghegan et al*.* [22]. Antibody information: TLR2 (AF2616-SP), Goat polyclonal (R&D Systems); TLR6 (NBP1-54336), Goat polyclonal (R&D Systems); IL-6Ra (MAB227), Mouse monoclonal IgG_1_, Clone 17506 (R&D Systems); gp130 (MAB2281), Mouse monoclonal IgG_1,_ Clone 29104 (R&D Systems); Vimentin (5741), Rabbit monoclonal, Clone D21H3 XP^*®*^ (Cell Signaling); E-cadherin (3195), Rabbit monoclonal, Clone 24E10^*®*^ (Cell Signaling); N-cadherin (13116), Rabbit monoclonal, Clone D4R1H XP^*®*^ (Cell Signaling); β-catenin (8480), Rabbit monoclonal, Clone D10A8 XP^*®*^ (Cell Signaling); β-actin (4970), Rabbit monoclonal (Cell Signaling); GAPDH (CB1001) Rabbit monoclonal (Calbiochem); HRP conjugated anti-goat IgG (705-035-003) (Jackson Immunolabs); HRP conjugated anti-rabbit IgG (W401)(Promega).

### Densitometry

Densitometric analyses of protein expression on western blots were performed using the Image Lab Software, Bio-Rad Laboratories.

### *Anoikis* assay (anchorage-independent assay)

The assay was performed was essentially as described by McNamee and O’Driscoll [23]. Tissue culture plates were uncoated or coated with poly-(hydroxyethyl methacrylic) acid (p-HEMA) thus inhibiting the ability of the cells to attach to the tissue culture plastic. 24-well plates were coated with 200 µl of 12 mg/ml poly-(hydroxyethyl methacrylic) acid (Sigma-Aldrich) in 95% ethanol or 95% ethanol as control for two consecutive days and dried overnight in a laminar flow hood at room temperature. DOK and SCC-4 cells were then seeded at 4 × 10^5^ cells/ml per well and allowed to attach overnight. Upon determination of the optimal antibody concentrations, DOK and SCC-4 cells were treated with 30 ng/ml rhIL-6, 10 μg/ml IL-6 neutralising mAb, 40 μg/ml IL-6Ra mAb, combination of 30 ng/ml rhIL-6 and 40 μg/ml IL-6Ra mAb, 200 ng/ml Pam2CSK4, combination of 200 ng/ml and 10 μg/ml anti-TLR2 neutralising antibody. Complete medium and IgG_2B_ isotype control for the IL-6 neutralising mAb were used as control. 24 h later, 50 µl of alamarBlue^*®*^ dye (Invitrogen, Carlsbad, California, USA) was added to each well and incubated again at 37 °C/5% CO_2_ for 3.5 h. Fluorescence was measured at excitation and emission wavelengths of 544 nm and 590 nm, respectively, using the Spectramax Gemini Plate Reader using SOFTmax Pro version 4.9 (Molecular Devices, Sunnyville, C.A, U.S.A.). Antibody information: Human anti-IL-6Ra (MAB227), Mouse monoclonal IgG_1_, clone 17506 (R&D systems); Human IL-6 (MAB2061), 1936 (R&D systems); Isotype Control (MAB004), mouse monoclonal IgG_2B_, clone 20116 (R&D systems); Human TLR2 antibody (HM1054), mouse monoclonal IgG_1,_ Clone T2.5 (Hycult Biotech).

### Statistical analysis

Statistical analyses were performed using the computer based mathematical package Graph Pad Prism 8.0 software. All results were expressed as mean ± standard error of the mean (SEM). For comparisons of two groups data were analyzed using a two-tailed unpaired student’s *t*-test*,* while for comparisons of more than two groups, data were analyzed using one-way or two-way ANOVA followed by Tukey’s or Sidak’s or Bonferroni’s multiple comparison tests were performed. For all comparisons, *p*-value of *p < 0.05, **p < 0.01, ***p < 0.001 were considered to be significant.

## Results

### Effect of TLR agonists on cytokine secretion from primary gingival keratinocytes (PGK), dysplastic oral keratinocytes (DOK) and oral squamous cell carcinoma cells (SCC-4)

**Fig. 1:**
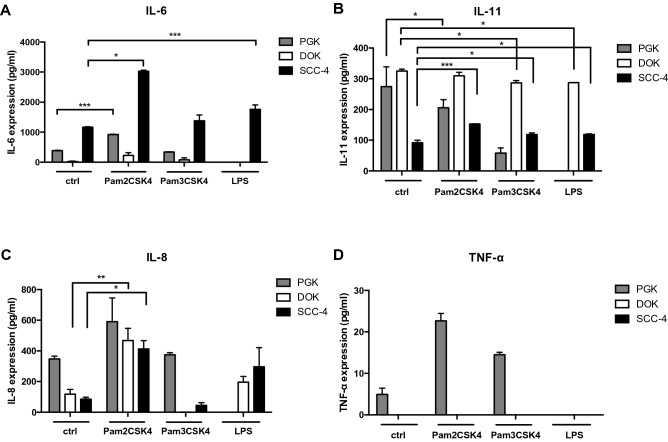
Cytokine production in PGK, DOK and SCC-4 cells. PGK, DOK and SCC-4 were untreated or stimulated for 24 h with TLR2 agonists: Pam3CSK4 (0.2 μg/ml), Pam2CSK4 (0.2 μg/ml) or TLR4 agonist LPS (0.5 μg/ml). Levels of (**A**) IL-6, (**B**) IL-11, (**C**) IL-8 and (**D**) TNF-α in the culture supernatants were determined by ELISA. Results were plotted using Graphpad prism 8. Data shown are representative of at least three independent experiments. Values represent the mean ± S.E.M of three independent experiments. Statistical analysis was performed using one-way ANOVA with a post *hoc* Tukey’s test to compare mean values between untreated and treated groups within the cell line, *p < 0.05; **p < 0.01; ***p < 0.001

Having optimized conditions for TLR ligand-dependent cytokine secretion (Supplemental Fig. S1), IL-6, IL-11, TNF-α and IL-8 secretion was determined, 24-h after TLR ligand stimulation of primary Gingival Keratinocytes (PGK), pre-cancerous Dysplastic Oral Keratinocytes (DOK) and cancerous Oral Squamous Cell Carcinoma 4 Cells (SCC-4). Figure 1A demonstrates that production of IL-6 was significantly higher from SCC-4 cells compared to PGK and DOK cells, regardless of the different ligand treatment. In particular, ELISA analysis revealed that the IL-6 secretion from normal untreated PGK cells (383.5 ± 15.7 pg/ml (3)) was significantly lower (threefold) than from untreated SCC-4 cells (1162 ± 18.2 pg/ml (3)) but significantly higher compared to DOK cells (20.06 ± 15 pg/ml (3)), while IL-6 release was almost undetectable. Furthermore, after Pam2CSK4 stimulation both PGK and SCC-4 cells increased IL-6 production threefold (917.7 ± 15.3 pg/ml (3) and 3026 ± 34.7 pg/ml (3), respectively), while DOK cells exhibited significantly lower IL-6 secretion (225.7 ± 94 pg/ml (3)) compared to both normal and cancerous cell line. No significant differences were observed after Pam3CSK4 stimulation in all treated groups when compared to their untreated controls (PGK: 338.5 ± 15.7 pg/ml (3), DOK: 79.04 ± 66.4 pg/ml (3), SCC-4: 1378 ± 193.5 pg/ml (3)), while LPS stimulation was found to significantly increase IL-6 release only from SCC-4 cells (1760 ± 148.7 pg/ml (3)). Interestingly, no release of IL-6 was found from PGK and DOK cell lines after LPS stimulation suggesting that this cytokine is not present in their supernatant, (mean ± SEM (*n*)).

Regarding the secretion of another member of the IL-6 family, namely IL-11 (Fig. 1B), DOK cells produced the highest amount of IL-11 release compared to PGK and SCC-4, regardless of the different treatments received. In other words, no effect of the ligands was observed between the Pam2CSK4 stimulated DOK cells (Pam2CSK4: 309.9 ± 11.4 pg/ml (3), *p* = 0.52), when compared with the untreated DOK cells (325.3 ± 6.4 pg/ml (3)), while a significant decrease was observed after Pam3CSK4 (286.9 ± 7.6 pg/ml (3), *p* = 0.03) and LPS (287.3 ± 0.2 pg/ml (3), *p* = 0.03) stimulation. On the contrary, IL-11 secreted from untreated SCC-4 cells (92.2 ± 7.5 pg/ml (3)) was significantly lower than that from untreated PGK (274.6 ± 64.2 pg/ml (3)) and DOK cells (325.3 ± 6.4 pg/ml (3)). Interestingly, IL-11 was shown to be significantly increased after stimulation with Pam2CSK4, Pam3CSK4 and LPS ligand in SCC-4 cells (152.7 ± 0.7 pg/ml (3), *p* < 0.0001, 118.3 ± 5.1 pg/ml (3), *p* = 0.02 and 118.9 ± 2.1 pg/ml (3), *p* = 0.02, respectively), while Pam3CSK4 stimulation decreased IL-11 secretion almost fivefold from PGK cells compared to the untreated control group. Pam2CSK4 stimulation did not show any effect on IL-11 release in PGK cells (205.8 ± 26 pg/ml (3)), while no release of IL-11 was observed from the same cell line after LPS stimulation suggesting that this cytokine is not present in the medium (mean ± SEM (*n*)).

Untreated PGK cells secreted the highest IL-8 levels (347.6 ± 18.7 pg/ml (3)) compared to untreated DOK (118.2 ± 30.8 pg/ml (3)) and untreated SCC-4 cells (85.5 ± 12.2 pg/ml (3)) (Fig. 1C). After stimulation with Pam2CSK4, all cells increased IL-8 release. In particular, DOK (467.6 ± 80.2 pg/ml (3), *p* = 0.005) and SCC-4 cells (412.9 ± 54.4 pg/ml (3), *p* = 0.038) exhibited a significant increased secretion compared to their untreated groups, while Pam2CSK4 treated PGK cells (591.2 ± 154.5 pg/ml (3), *p* = 0.2) slightly but not significantly increased IL-8 production. Furthermore, after Pam3CSK4 stimulation, no significant difference was detected between the treated (374.1 ± 14.6 pg/ml (3), *p* = 0.99) and untreated control group (347.6 ± 18.7 pg/ml (3)) in PGK cells, while IL-8 production was almost undetectable in both DOK (0.85 ± 0.5 pg/ml (3)) and SCC-4 cells (45.2 ± 16.8 pg/ml (3)). All cells were also treated with LPS, although no significant difference was found between the untreated and treated groups in both DOK (196.2 ± 37.8 pg/ml (3), *p* = 0.7) and SCC-4 (297.2 ± 123.6 pg/ml (3), *p* = 0.2), while IL-8 was not detected in LPS-treated PGK cells, suggesting that this cytokine is not produced (mean ± SEM (*n*)). Figure 1D shows that production of the cytokine TNF-α was barely detectable from PGK cells and undetectable from both DOK and SCC-4 cells, regardless of the ligand stimulation received. There were no significant differences among untreated and treated groups, suggesting that this cytokine is not secreted.

### Effect of anti-TLR2 blocking antibody on cytokine production and TLR2/TLR6 protein expression levels in DOK and SCC-4 cells

**Fig. 2:**
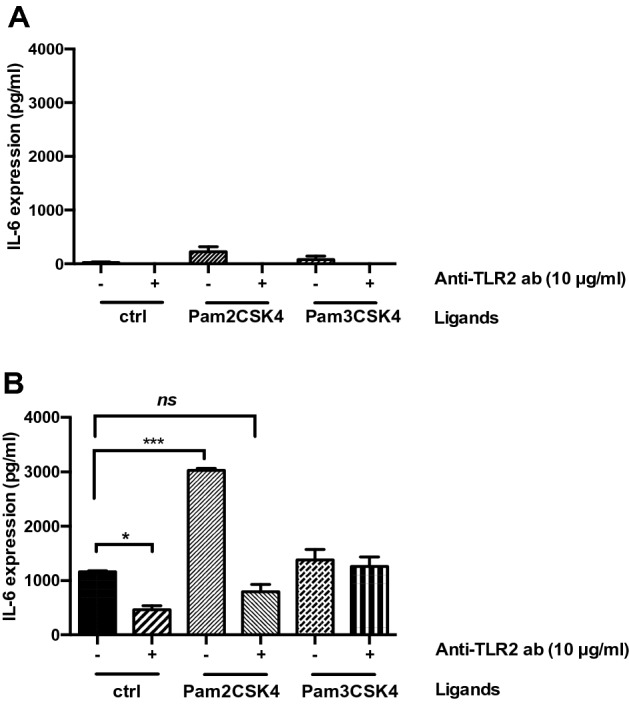
The effect of TLR2 neutralising antibody on IL-6 production in SCC-4 cells. (**A**) DOK and (**B**) SCC-4 cells were untreated or pre-treated with anti-TLR2 Ab (10 μg/ml) for 1 h, prior to stimulation with Pam2CSK4 (0.2 μg/ml) and Pam3CSK4 (0.2 μg/ml) for 24 h. Culture supernatants were analysed for IL-6 secretion by ELISA. Results were plotted using Graphpad prism 8. Values represent the mean ± S.E.M of three independent experiments. Statistical analysis was performed using one-way ANOVA with a post *hoc* Dunnett’s test to compare mean values among the groups, *p < 0.05; **p < 0.01; ***p < 0.001

Having established the optimal concentration of anti-TLR2 blocking antibody in DOK and SCC-4 cells (Supplemental Fig. S2), the effect of anti-TLR2 antibody (10 μg/ml) on IL-6 release was analysed in these cell lines (Fig. 2). DOK (Fig. 2A) and SCC-4 (Fig. 2B) cells were pre-treated with 10 μg/ml anti-TLR2 blocking antibody for 1 h prior to stimulation with TLR2 agonists (Pam2CSK4 and Pam3CSK4). Measurement of IL-6 production after 24 h demonstrated that anti-TLR2 significantly inhibits IL-6 secretion in SCC-4 cells, while no IL-6 release was detected in DOK cells in the groups treated with anti-TLR2 antibody or in combination with TLR2 ligands. In particular, anti-TLR2 blocking antibody significantly diminished IL-6 secretion (461 ± 73.9 pg/ml (3)) when it was administered alone, while when combined with Pam2CSK4, IL-6 secretion decreased (794.8 ± 134.1 pg/ml (3)) significantly compared to Pam2CSK4 treated group (3026 ± 34.7 pg/ml (3)) but not significantly different when compared to the untreated control (1162 ± 18.2 pg/ml (3)). Interestingly, there was no effect of Pam3CSK4 alone or in combination with anti-TLR2, on IL-6 production when compared to the untreated control for SCC-4 cells (mean ± SEM (*n*)).

**Fig. 3:**
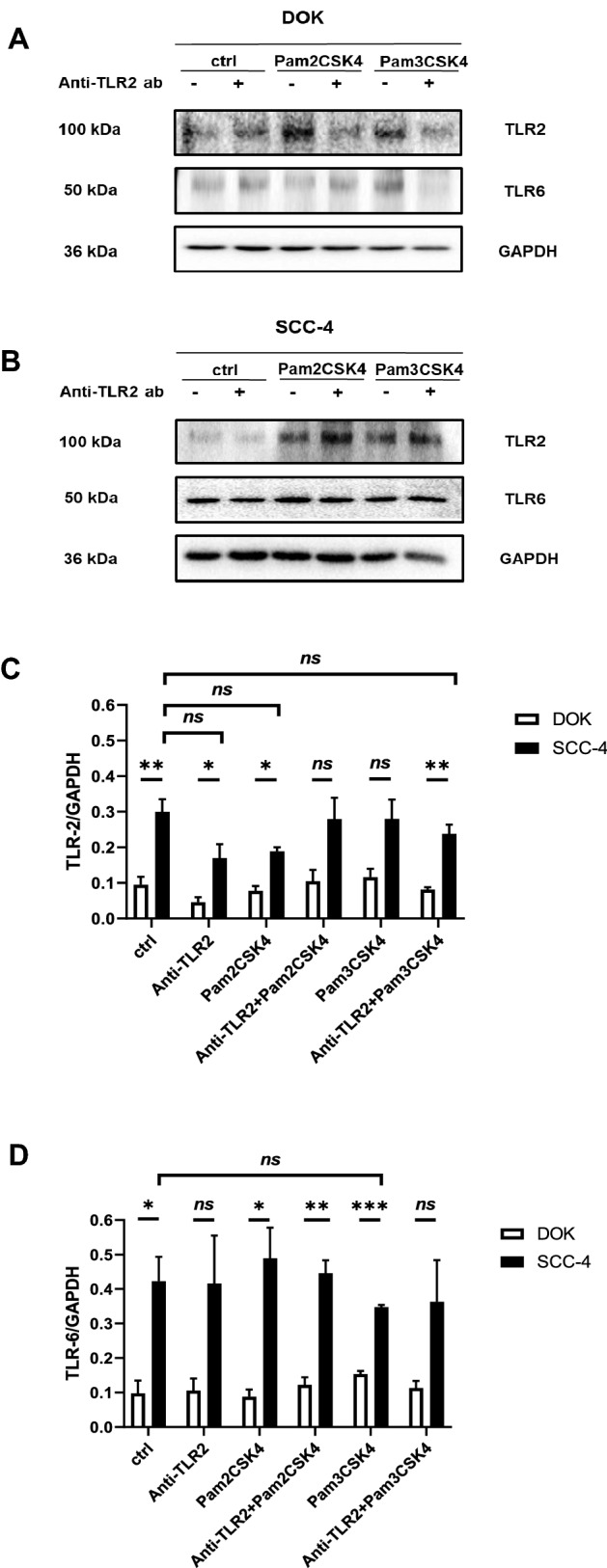
TLR2 and TLR6 expression levels in SCC-4 cells and DOK cells. DOK and SCC-4 cells were left untreated or pre-treated with anti-TLR2 Ab (10 μg/ml) for 1 h, prior to stimulation with Pam3CSK4 (0.05 μg/ml) or Pam2CSK4 (0.05 μg/ml) for 24 h. Lysates were collected and samples were run on 12% SDS-PAGE gel. Western Blot were probed with anti-TLR2 and anti-TLR6 antibodies with anti-GAPDH used as a loading control. Experiment was done in triplicate. Densitometric analysis was performed using image lab software on TLR2, TLR6 and GAPDH blots. Sample blots for (**A**) DOK and (**B**) SCC-4 and collated densitometry data for (**C**) TLR2 and (**D**) TLR6 are presented. Results were plotted using Graphpad prism 8. Values represent the mean ± S.E.M of three independent experiments. Statistical analysis was performed using two-way ANOVA with a post *hoc* Sidak’s test to compare mean values among the groups, *p < 0.05; **p < 0.01; ***p < 0.001

Based on the aforementioned TLR2 ligand-dependent observations, immunoblot analysis was used to determine protein expression levels of TLR2 and TLR6 in both DOK and SCC-4 cells (Fig. 3A, B). Collated data revealed that TLR2 expression (Fig. 3C) is significantly higher in untreated SCC-4 cells compared to untreated DOK cells. Interestingly, no significant downregulation of TLR2 was observed when both cell lines were treated with anti-TLR2 or anti-TLR2 in combination with TLR2 ligands (Pam2CSK4 and Pam3CSK4) when compared to their untreated controls. Furthermore, TLR6 expression (Fig. 3D) is also significantly higher in untreated SCC-4 compared to DOK cells, but there was no effect on TLR expression with addition of anti-TLR2 blocking antibody alone or in combination with TLR ligands (Pam2CSK4 and Pam3CSK4) in either DOK or SCC-4 cells.

### IL-6Ra and gp130 protein expression and effect of anti-TLR2 blocking antibody on IL-6 production in DOK and SCC-4 cells

**Fig. 4:**
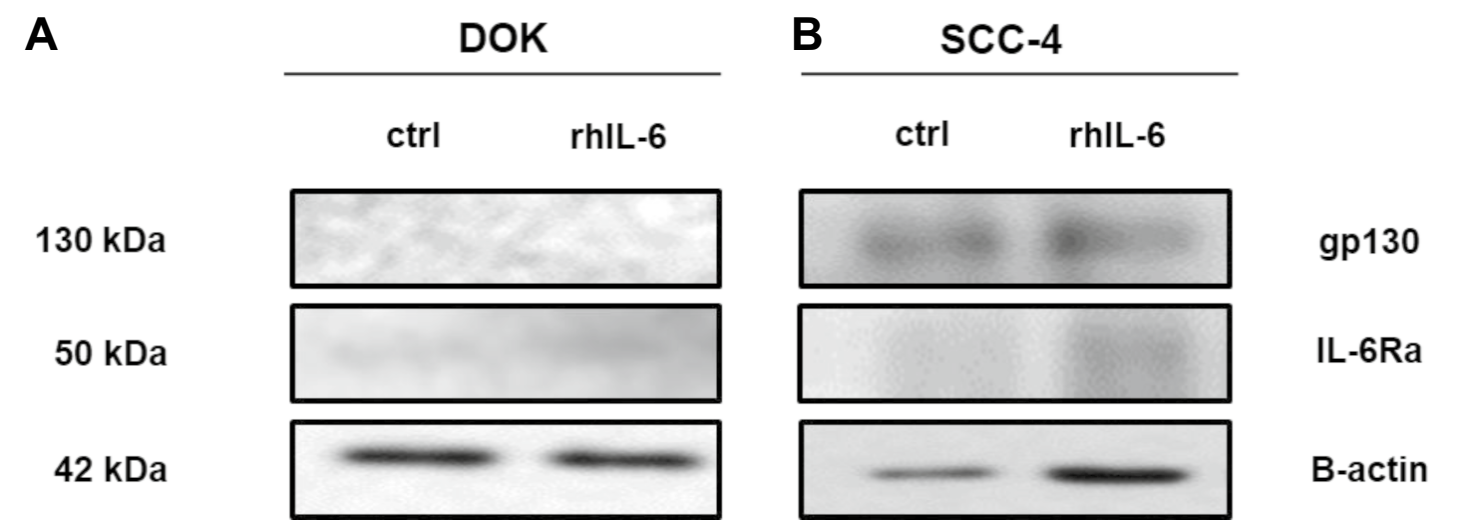
gp130 and IL-6Ra expression in SCC-4 cells and DOK cells. DOK and SCC-4 cells were untreated or treated with rhIL-6 (30 ng/ml) for 24 h. Lysates were collected and samples were run on 8% SDS-PAGE gel. Western Blot were probed with anti-gp130 and anti-IL-6Ra antibody with anti-β-actin used as a loading control. Experiments was done in triplicate. Densitometric analysis was performed using image lab software on gp130, IL-6Ra and β-actin blots. Sample blots for (**A**) DOK and (**B**) SCC-4 and collated densitometry data for (Fig. S5A) gp130 and (Fig. S5B) IL-6Ra are presented. Results were plotted using Graphpad prism 8. Values represent the mean ± S.E.M of three independent experiments. Statistical analysis was performed using one-way ANOVA with a post *hoc* Sidak’s test to compare mean values among the groups, *p < 0.05; **p < 0.01; ***p < 0.001. Western blot figures represent one representative result

Immunoblot analysis revealed that both gp130 and IL-6Ra are more prominent in SCC-4 cells compared to DOK cells. Figure 4A and B displays one representative immunoblot. Collated data in Supplemental Fig. S3 shows that (A) gp130 and (B) IL-6Ra expression is significantly greater in SCC-4 cells compared to DOK cells, and that rhIL-6 treatment had no effect on expression levels.

### A comparison of epithelial to mesenchymal transition (EMT) profile of dysplastic oral keratinocytes (DOK) cells and immortal squamous cell carcinoma 4 (SCC-4) cells

In order to determine the EMT profile in DOK and SCC-4 cells, immunoblot analysis was employed and cells were examined untreated and treated with 30 ng/ml rhIL-6, 10 μg/ml IL-6 monoclonal neutralising antibody, or 40 μg/ml IL-6Ra monoclonal antibody for 24 h. Cells were collected, lysed and ran on 8% SDS-PAGE gel before being transferred to PVDF membrane and probed using antibodies for E-cadherin, N-cadherin, β-catenin and vimentin. An anti-GAPDH antibody was used as a loading control. Both cell lines were treated with 10 μg/ml of an IgG isotype antibody for 24 h as a control for the IL-6 monoclonal neutralising antibody. Examples of these immunoblots for DOK cells are given in Supplemental Fig. S4A and B for SCC-4 cells and display one representative experiment of untreated and treated with 10 μg/ml IgG isotype control, 30 ng/ml rhIL-6, 10 μg/ml IL-6 neutralising monoclonal antibody and 40 μg/ml IL-6Ra monoclonal antibody.

**Fig. 5:**
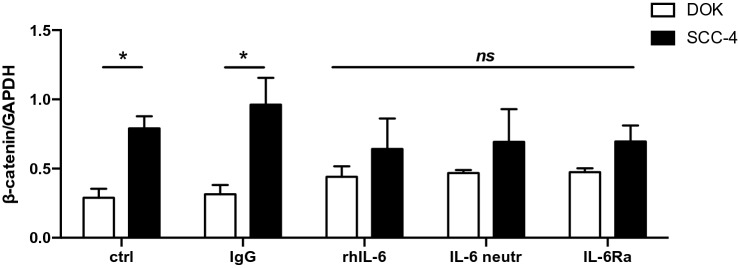
EMT protein expression in SCC-4 cells and DOK cells. DOK and SCC-4 cells were untreated or treated with 10 μg/ml IgG isotype control, 30 ng/ml rhIL-6, 10 μg/ml IL-6 neutralising antibody, and 40 μg/ml IL-6Ra antibody for 24 h. Lysates were collected and samples were run on 8% SDS-PAGE gel. Sample blots for DOK and SCC-4 and collated densitometry data for β-catenin is presented. β-catenin expression is significantly upregulated in SCC-4 untreated group compared to DOK untreated counterparts, while (Fig. S6C) E-cadherin is not expressed in SCC-4 cells, (Fig. S6D) N-cadherin and (Fig. S6E) vimentin protein expression is absent in DOK cells. Results were plotted using Graphpad prism 8. Results represent three experiments performed (mean ± SEM (*n*)). Statistical analysis was performed using two-way ANOVA to compare mean values among the groups, *p ≤ 0.05, **p ≤ 0.01, ***p ≤ 0.001

As shown significant upregulated β-catenin was found in untreated control SCC-4 cells compared to untreated control DOK cells (Fig. 5), while no Ε-cadherin was detected in SCC-4 cells (Supplemental Fig. S4C). Interestingly, both N-cadherin (Supplemental Fig. S4D) and vimentin (Supplemental Fig. S4E) were upregulated in SCC-4 cells, however no expression of the aforementioned proteins was shown in DOK cells respectively). Furthermore, no differences were detected in the expression of the proteins examined, regardless of the treatments received (rhIL-6, IL-6 monoclonal neutralising antibody and IL-6Ra monoclonal antibody) compared to the untreated and isotype controls in either DOK or SCC-4 cells.

### A comparison of IL-6-dependent *anoikis* resistance of dysplastic oral keratinocytes (DOK) cells and immortal squamous cell carcinoma 4 (SCC-4) cells

**Fig. 6:**
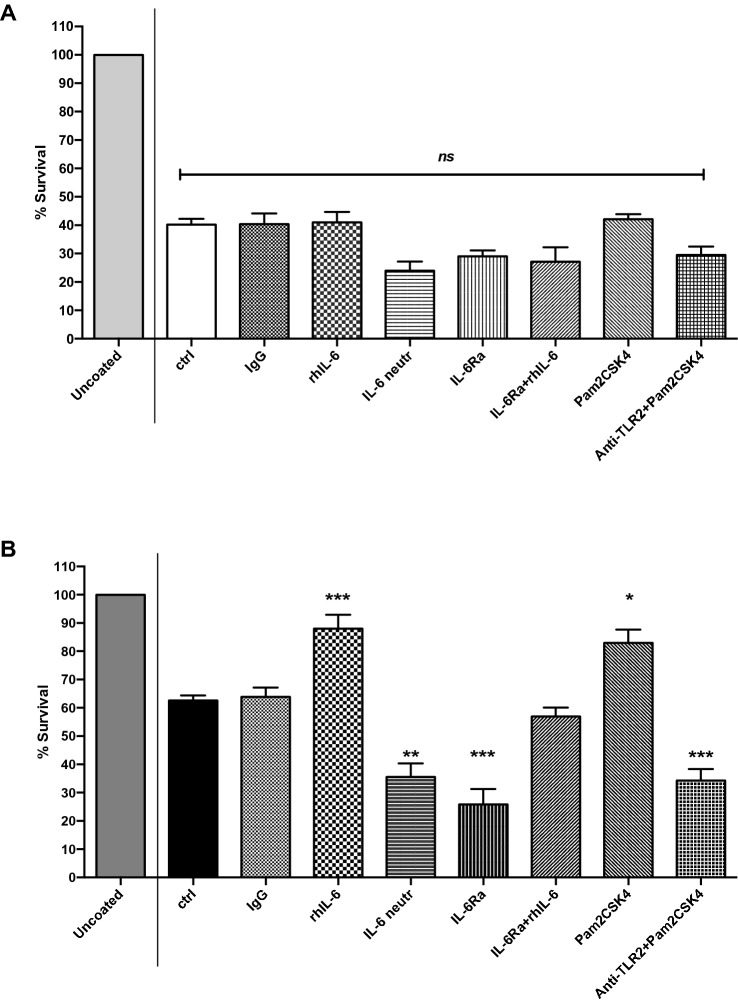
Survival rate in anoikis assays DOK and SCC-4 cells treated with rhIL-6, IL-6 neutralising antibody, IL-6Ra antibody, IL-6Ra + rhIL-6, Pam2CSK4, anti-TLR2 neutralising antibody + Pam2CSK4, and anti-TLR2 neutralising antibody + Pam2CSK4 for 24 h. Tissue culture plates were coated with 200 μl poly-(hydroxyethyl methacrylic) acid (poly-HEMA) thus inhibiting the ability of the cells to attach to the tissue culture plastic. (**A**) DOK and (**B**) SCC-4 cells were then seeded at 100,000 cells/well and allowed to attach. For those cells to be treated with 30 ng/ml rhIL-6, 10 μg/ml IL-6 neutralising antibody, 40 μg/ml IL-6Ra antibody, 40 μg/ml IL-6Ra + 30 ng/ml rhIL-6, 0.2 μg/ml Pam2CSK4, and 10 μg/ml anti-TLR2 neutralising antibody + 0.2 μg/ml Pam2CSK4, complete medium was used as control, as well as medium containing 10 μg/ml isotype IgG antibody. After 24 h, 50 μl of alamarBlue^®^ dye was added to each well and incubated again at 37 °C/5% CO_2_ for 3.5 h. SCC-4 cells demonstrated significant resistance to anoikis (% survival) when treated with 30 ng/ml rhIL-6 and 0.2 μg/ml Pam2CSK4 compared to the control group. There is a100% survival of adherent cells in the uncoated plates. Fluorescence was measured using a SpectraMax Gemini plate reader at excitation wavelength 544 nm and emission wavelength 590 nm. Results were plotted using Graphpad prism 8. Results represent at least three experiments performed in duplicate (mean ± SEM (*n*)). Statistical analysis was performed one-way ANOVA with a post hoc Tukey’s test to compare mean values among the groups, *p ≤ 0.05, **p ≤ 0.01, ***p ≤ 0.001

Metastatic cancer cells are able to survive and resist *anoikis* (evade apoptosis) [24]. One can mimic this situation in vitro by coating cell culture plates with poly(hydroxyethyl methacrylic) acid (poly-HEMA) thus inhibiting the ability of cells to adhere to the plate. In order to validate that *anoikis* resistance is a form of apoptosis in DOK and SCC-4 cells, adherent and in suspension (poly-HEMA), cells were examined by flow cytometry analysis of annexin V/PI-stained cells. Cells grown under anchorage—independent conditions (poly-HEMA) were observed to have induced apoptotic cells death compared to necrotic cells. DOK cells (poly-HEMA) were found to have the highest apoptotic rate (Supplemental Figs S5, S6). In this way, using the *anoikis* resistance assay, or anchorage-independent growth assay, survival rate of detached DOK and SCC-4 cells were examined from their artificial substratum. The *anoikis* resistance for the untreated groups was significantly lower in DOK (Fig. 6A) compared to SCC-4 cells (Fig. 6B) (40.2% ± 1.9 (9) and 62.6% ± 2.1 (7), respectively), and no difference was observed in *anoikis* survival rate upon treatment with IgG isotype antibody in either DOK or SCC-4 cells (40.3% ± 3.8 (5) and 63.8% ± 3.3 (5), respectively). Interestingly, the *anoikis* survival rate was significantly enhanced in SCC-4 cells after rhIL-6 addition and Pam2CSK4 addition (88% ± 4.9 (6) and 82.9% ± 4.8 (3), respectively), while no significant effect of rhIL-6 addition (40.9% ± 3.7 (9)) or Pam2CSK4 addition (42.1% ± 1.8 (5) was observed, compared to untreated and isotype controls, in DOK cells. However, *anoikis* survival rate was significantly diminished upon treatment of SCC-4 cells with monoclonal IL-6 neutralising and monoclonal IL-6Ra antibodies (35.6% ± 4.7 (3) and 25.8% ± 5.5 (3), respectively), while no significant differences were observed in DOK cells treated with IL-6 neutralising and IL-6Ra antibodies (24% ± 3.3 (3) and 29.1% ± 2.2 (3), respectively). No major effect was observed upon treatment with monoclonal IL-6Ra in combination with exogenous rhIL-6 in both DOK and SCC-4 cells (27.2% ± 5.1 (3) and 56.9% ± 3.2 (3), respectively), while combined treatment with Pam2CSK4 and anti-TLR2 neutralising antibody significantly decreased the survival rate in SCC-4 cells (34.3% ± 4.1 (5)), while no major change was shown again in DOK cells (29.5% ± 2.9 (4)).

## Discussion

Of the cytokines investigated, IL-6 was the most abundant cytokine produced by our cancerous cells which led us to give it some focus in this study. It was demonstrated that IL-6 secretion is threefold higher from SCC-4 cells compared to normal PGK, while IL-6 secretion is undetectable from DOK cells. This observation is consistent with that of Chen et al*.* [25] who observed similar amount of elevated IL-6 production in SCC-4 cells compared to another oral squamous cell carcinoma, SCC-25 cells, which showed threefold lower IL-6 production. Another study [26] also suggested enhanced production of salivary IL-6 as a diagnostic marker for leukoplakia and OSCC cells. Furthermore, clinical studies have reported increased IL-6 serum levels and salivary IL-6 concentration in OSCC patients compared to patients with leukoplakia and healthy controls, suggesting that IL-6 could be a crucial biomarker in HNSCC diagnosis [27, 28].

Our investigation progressed to determine whether TLR ligand activation had an effect on IL-6 production levels from the cells used in this study. When SCC-4 cells were treated with the TLR2/TLR6 agonist, Pam2CSK4, there was a significant threefold increase in IL-6 production in both SCC-4 and PGK cells. No significant increase in IL-6 production was observed with TLR1/TLR6 ligand, Pam3CSK4. Interestingly, Chuang et al*.* [29] examined the intracellular signalling pathway involved in IL-6-induced intracellular adhesion molecule-1 (ICAM-1) expression and tumour migration in SCC-4 cells by activating a number of crucial pathways, and a possible role for IL-6 in oral cancer metastasis was suggested. It is noteworthy that when SCC-4 cells were treated with the TLR4 agonist, LPS, IL-6 secretion levels significantly increased but not to the same extent as with TLR2/TLR6 agonist, Pam2CSK4. However, there is evidence in the literature that lipopolysaccharide (LPS) is able to induce TLR4-mediated epithelial–mesenchymal transition and cell migration in SCC-4 cells [30] which may or may not be due to IL-6.

By contrast, it was revealed that both PGK and DOK cells IL-11 secretion levels were threefold higher when compared to SCC-4 cells. Interestingly, only Pam3CSK4 and LPS agonists slightly but significantly affected IL-11 secretion levels in DOK cells, while only Pam3CSK4 significantly diminished IL-11 production in PGK cells. Significant IL-11 increase was also found in SCC-4 cells after stimulation with all agonists but in general IL-11 levels were significantly lower than the levels in treated dysplastic counterparts. Elevated levels of IL-11 (~ 250 μg/l) have been identified in breast cancer patients which is associated with bone metastasis [31].

TNF-α, a widely expressed pleiotropic cytokine, is increased during inflammation and cancer in saliva and patient sera. Indeed, it has been reported that TNF-α levels might act as diagnostic markers for detection of oral cancer since it is more abundant in saliva than in plasma [32, 33] and Scheff et al. [34] demonstrated significant secretion of TNF-α from certain oral squamous cancer cells in a pain model. However, in our in vitro system, TNF-α secretion levels were undetectable regardless of the stimulations received in DOK and SCC-4 cells, while negligible detection of TNF-α production was observed in normal PGK cells.

IL-8 has been found in the tumour microenvironment in a range of different cancer types, including OSCC tumours. Clinical studies have reported elevated salivary IL-8 levels in OSCC patients [35, 36] compared to healthy controls. However, the results in this study did not reveal substantial IL-8 production in SCC-4 cells.

TLRs have been reported to be expressed in various types of cancers with key roles in carcinogenesis and tumour progression [37]. Since Pam2CSK4, a TLR2/6 agonist, was the ligand that induced the most IL-6 secretion, and to a lesser extend IL-8 and IL-11 cytokine production in SCC-4 cells, TLR2 neutralising antibody combined with either Pam2CSK4 or Pam3CSK4 were used to further examine their effect on IL-6 production in DOK and SCC-4 cells. It was demonstrated that treatment with anti-TLR2 neutralising antibody resulted in a twofold decrease in IL-6 production from SCC-4 cells, while IL-6 was undetectable from pre-cancerous DOK cells, and no effect of anti-TLR2 neutralising antibody could be detected. Furthermore, no effect on the IL-6 production was observed after treatment with TLR2 neutralising antibody combined with Pam3CSK4, which led us to postulate that TLR1 is probably not expressed to any great degree in either DOK or SCC-4 cells.

In support of these findings, a previous report revealed higher TLR2 expression in the microenvironment of the keratinocytes of dysplastic epithelium and OSCC, when compared to hyperplasic cells [38]. Interestingly, a report by Ikehata et al. [39] identified, by western blot and immunohistochemistry, enhanced expression of TLR2, TLR1 and TLR6 in human OSCC tissue compared to adjacent non-malignant tissue, as well as significant amount of TLRs in a range of OSCC cells (HSC3, HSC3-M3, SCC-9, SCC-25 cells). The data in this study are consistent with these observations, in that TLR2 and TLR6 were found significantly upregulated in SCC-4 cells compared to DOK cells.

After observing the effect of TLR2/6 ligand on IL-6 secretion from DOK and SCC-4 cells and the levels of TLR2 expression in these cells, it was decided to investigate whether there was expression of receptors for IL-6, or more particularly expression of IL-6Ra and gp130, in DOK and SCC-4 cells using western blotting. These findings suggest significantly higher IL-6Ra and gp130 expression in SCC-4 cells compared to DOK cells, regardless of the stimulation with rhIL-6. This result suggests that classical IL-6 signaling occurs in SCC-4 cells since both receptors were expressed, and consequently IL-6 is able to bind to the membrane-bound receptor IL-6Rα, which has the capacity for heterodimerization with gp130 and thus forming a complex that allows the JAK/STAT signaling cascade to operate [40].

The effect of endogenous and exogenous IL-6 on cancer phenotypes of pre-cancerous human tongue DOK cells and cancerous SCC-4 human tongue cells were also investigated. *Anoikis* resistance, markers of EMT, and cell death were characterized in these cells. Evidence is provided for IL-6 playing a central role in *anoikis* resistance. Data from this study show that DOK cells displayed non-EMT characteristics, expressing E-cadherin and lower levels of β-catenin compared to SCC-4 cells. High levels of vimentin and N-cadherin were observed in SCC-4 cells compared to DOK cells, while complete loss of E-cadherin was demonstrated in SCC-4 cells, confirming the EMT phenotype of these cells. Moreover, it was demonstrated by Wu et al*.* [41] that IL-6 induced EMT-related gene expression, leading to EMT phenotypes in pancreatic cancer cell (AsPC-1, BxPC-3, and Panc-1 cells), in which mesenchymal-like markers including N-cadherin and vimentin were upregulated, and epithelial-like marker, E-cadherin were downregulated. Interestingly, E-cadherin was upregulated while N-cadherin and vimentin were downregulated upon treatment with IL-6 neutralising antibody in these pancreatic cells [41]. Similarly, decreased expression of E-cadherin and increased vimentin was observed in response to IL-6 in osteosarcoma cells (U2OS and MG-63 cells), while those effects were reversed after treatment with small interferring-IL-6, suggesting a potential implication of IL-6 in acquisition/maintenance of stemness properties [42]. Another study has reported that activation of IL-6 signalling is correlated with aggressive tumour behaviour and EMT changes in pharyngeal cancer [43], lymph node metastasis and disease recurrence [25], while activated IL-6Ra/gp130 signalling induced various pathways

associated with the regulation of tumour proliferation and metastasis [44]. However, in this study, there was no effect of rhIL-6 addition, addition of IL-6 neutralising antibody or addition of IL-6Ra monoclonal antibody on β-catenin, E-cadherin, N-cadherin or vimentin expression levels in either DOK or SCC-4 cells, suggesting that EMT profile is not regulated by IL-6 in these cells.

*Anoikis* resistance, or the ability for cells to live detached from the extracellular matrix, is a property of epithelial cancers. In this study, resistance to *anoikis* was assessed in DOK and SCC-4 cells. The results demonstrated that SCC-4 cells showed significantly greater resistance to *anoikis* when compared to DOK cells. In particular, half the population of untreated dysplastic DOK cells (49.2%) were able to resist *anoikis* when detached from the ECM, while survival rates of untreated cancerous SCC-4 cells were significantly higher (1.5-fold at 76.5%) as might be expected from cancerous cells. It was then decided to look at the effect of IL-6 on *anoikis* resistance in DOK and SCC-4 cells. The results indicated that rhIL-6 treatment significantly enhanced (1.4-fold) resistance to *anoikis* in SCC-4 cells when compared to untreated control levels, an observation consistent with that seen by Fofaria and Srivastava [15] for pancreatic cancer cells.

Similarly, *anoikis* resistance in SCC-4 cells was increased (1.3-fold) upon stimulation with Pam2CSK4, an observation that is consistent with a role for endogenous IL-6 being important for *anoikis*, and consistent with the observation that Pam2CSK4 triples IL-6 secretion from SCC-4 cells. Again, consistent with a role for endogenously produced IL-6 in *anoikis*, is the observation that treatment of SCC-4 cells with IL-6 receptor (IL-6Ra) monoclonal antibody significantly reduced (2.5-fold) *anoikis* resistance, a result that is further endorsed by the observation that neutralising IL-6 or blocking TLR2 with the monoclonal antibodies also reduces *anoikis* resistance in SCC-4 cells (1.8-fold). Consistent with these observations is the observation that *anoikis* resistance was not affected when extrinsic IL-6 was added in combination with IL-6Ra addition. There was no effect of rhIL-6 addition, IL-6Ra addition, IL-6 neutralizing antibody, TLR2 blocking antibody or a combination of rhIL-6 plus IL-6RA on *anoikis* resistance in pre-cancerous dysplastic DOK cells.

## Summary and conclusions

We show that a quantitatively significant amount of IL-6 is secreted by oral cancerous SCC-4 cells. In addition, the TLR2 ligand Pam2CSK4 enhances IL-6 secretion from SCC-4 cells and enhances *anoikis* resistance. The quantitatively large increase in IL-6 production from SCC-4 cells with addition of the Pam2CSK4, but not Pam3CSK4, is consistent with our data confirming the existence of TLR2/6 and IL-6Ra/gp130 receptors in SCC-4 cells. The fact that DOK cells do not produce IL-6 is consistent with the lack of IL-6Ra/gp130 receptors in DOK cells. The role of TLR2 on enhanced IL-6 production is further cemented by the observation that anti-TLR2 receptor blocking antibodies reduced, by half, the IL-6 production in SCC-4 cells and significantly reduces *anoikis* resistance. The central role played by IL-6 in *anoikis* in SCC-4 cells is also demonstrated by the observations that added recombinant IL-6 increases *anoikis* resistance, and that IL-6 neutralizing antibody and IL-6 receptor antibodies reduce *anoikis* resistance. This study provides evidence, for the first time, that IL-6 drives *anoikis* resistance in SCC-4 cells.

## Supplementary Information

Below is the link to the electronic supplementary material.

Supplementary file1 (PDF 785 KB)

## Abbreviations

DCBM: Dermal cell basal medium
DOK: Dysplastic oral keratinocytes
ECM: Extracellular matrix
ELISA: Enzyme linked immunoassay
EMT: Epithelial to mesenchymal transition
FBS: Fetal bovine serum
GAPDH: Glyceraldehyde-3-phosphate dehydrogenase
HRP: Horseradish peroxidase
IgG: Gamma immunoglobin
IL: Interleukin
IL-6Ra: Interleukin-6 receptor antibody
rhIL-6: Recombinant human interleukin 6
LPS: Lipopolysaccharide
OSCC: Oral squamous cancer cells
PGK: Primary gingival keratinocytes
poly-HEMA: Poly(hydroxyethyl methacrylic) acid
SDS-PAGE: Sodium dodecyl sulphate polyacrylamide gel electrophoresis
SEM: Standard error of the mean
TLR: Toll-like receptor
TNF: Tumour necrosis factor
