# Supplementary material for: Evidence of a role for interleukin-6 in anoikis resistance in oral squamous cell carcinoma
Source: Med Oncol. 2022 Apr 29;39(6):60. doi: 10.1007/s12032-022-01664-5 (PMC9050791; doi:10.1007/s12032-022-01664-5)

Supplemental Figure S1

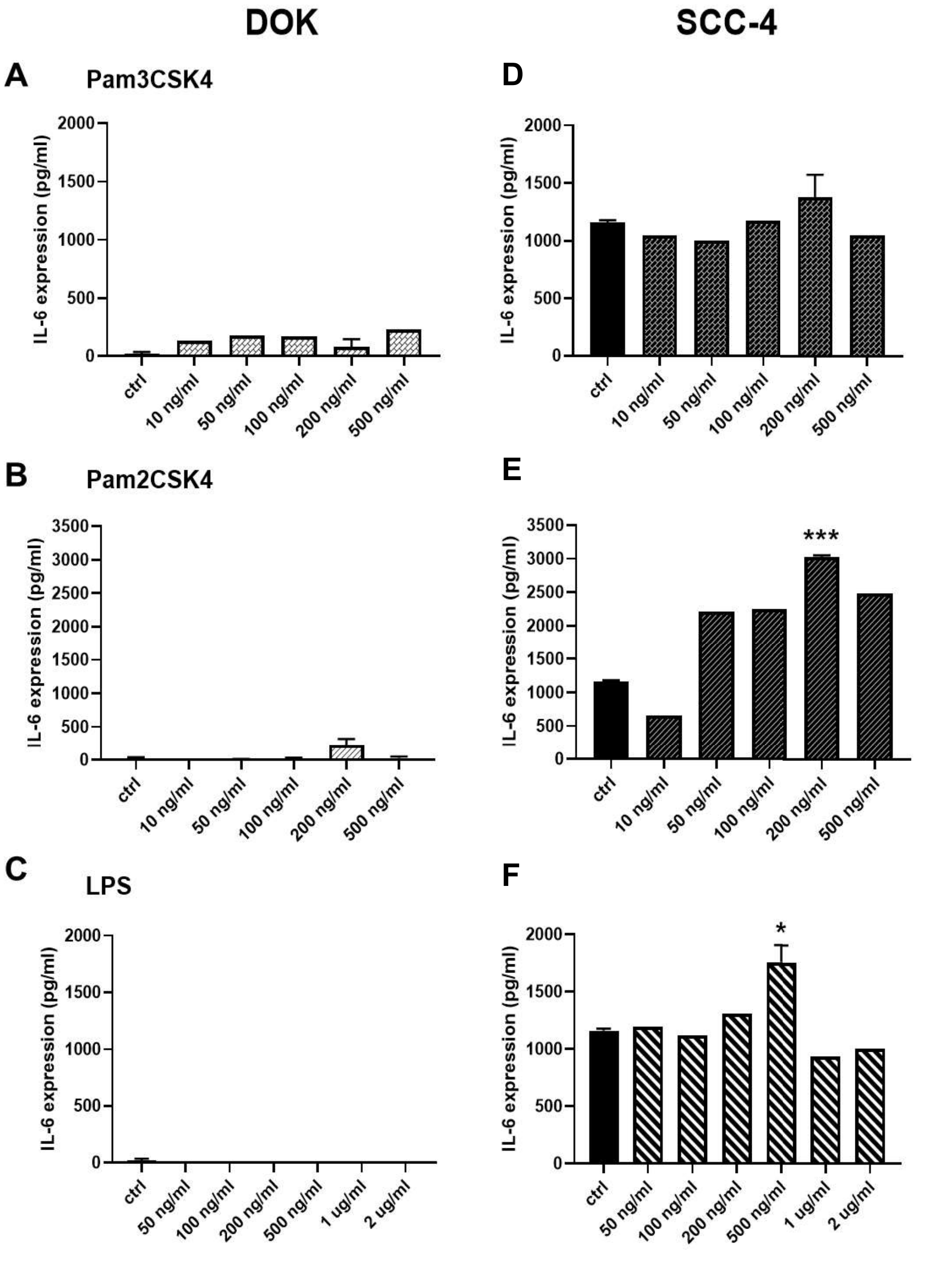

Supplemental Figure S2

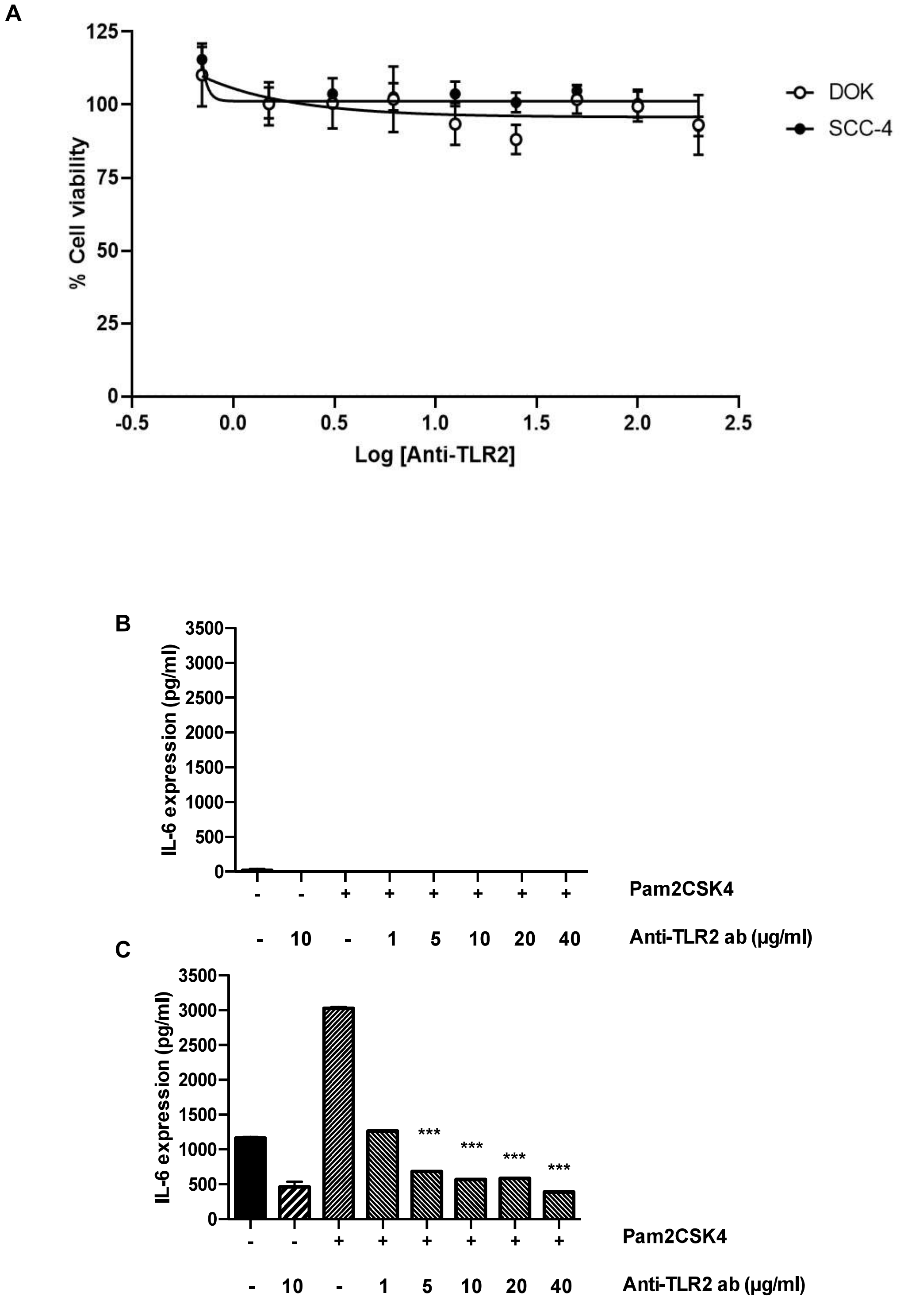

Supplementary Figure S3

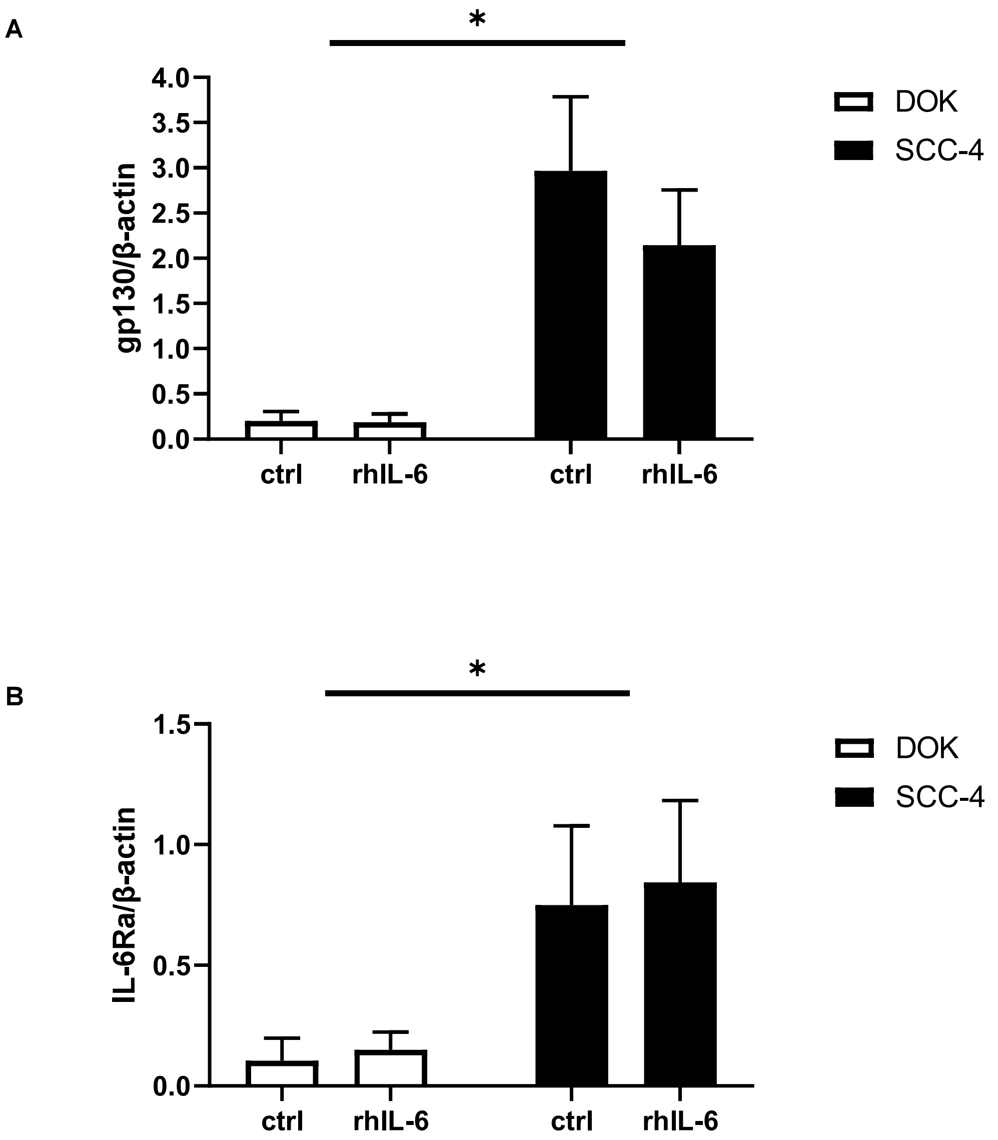

Supplementary Figure S4

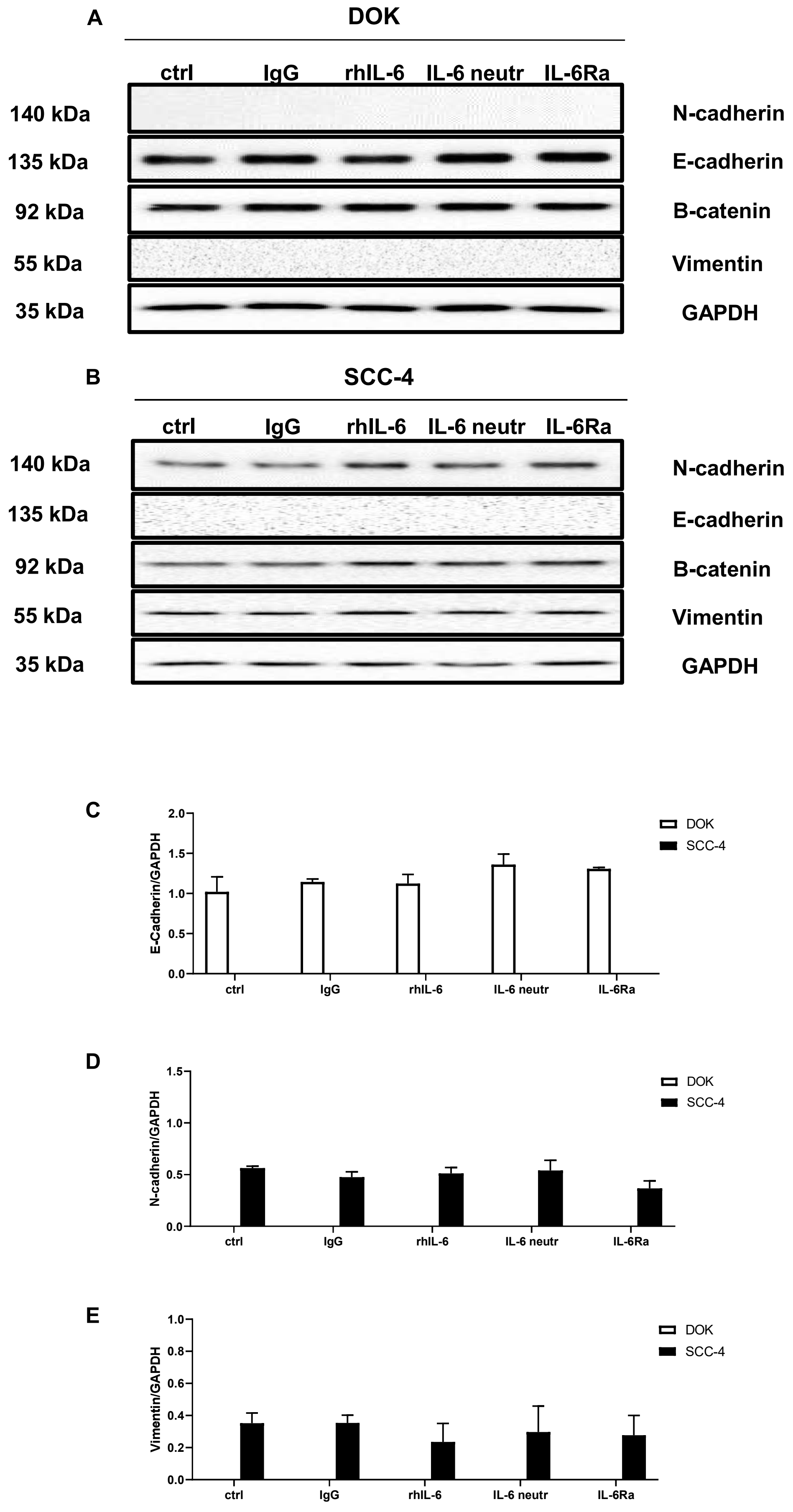

Supplementary Figure S5

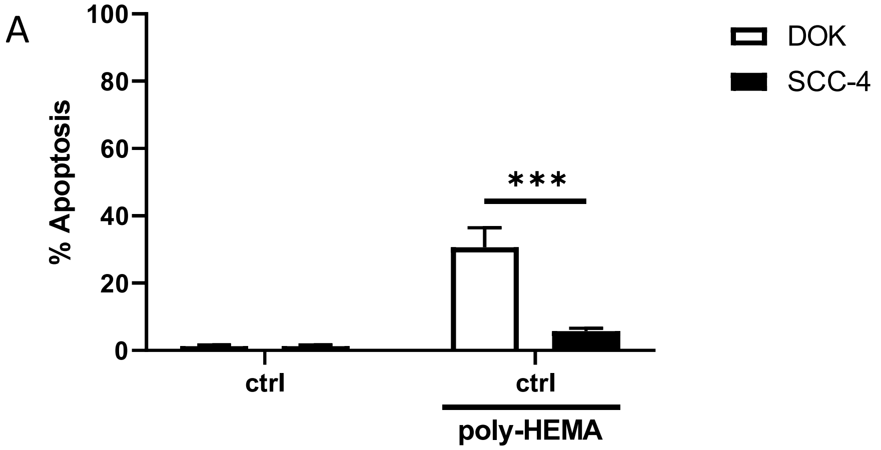

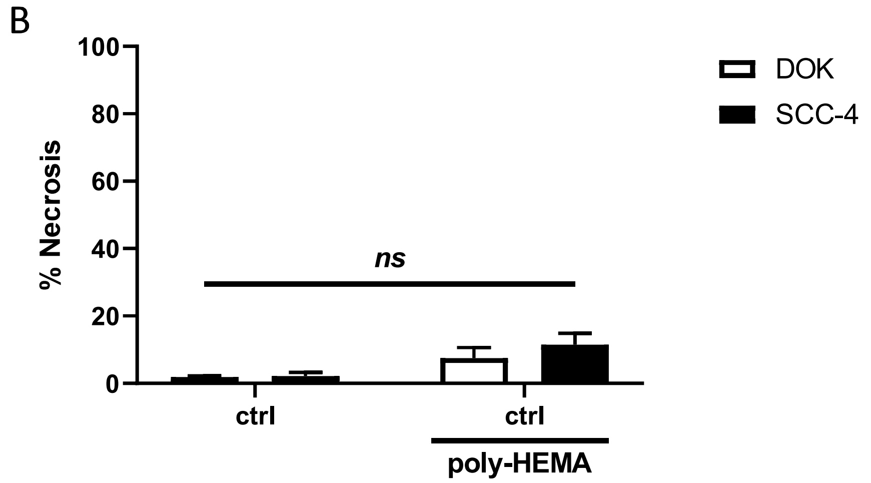

Supplementary Figure S6

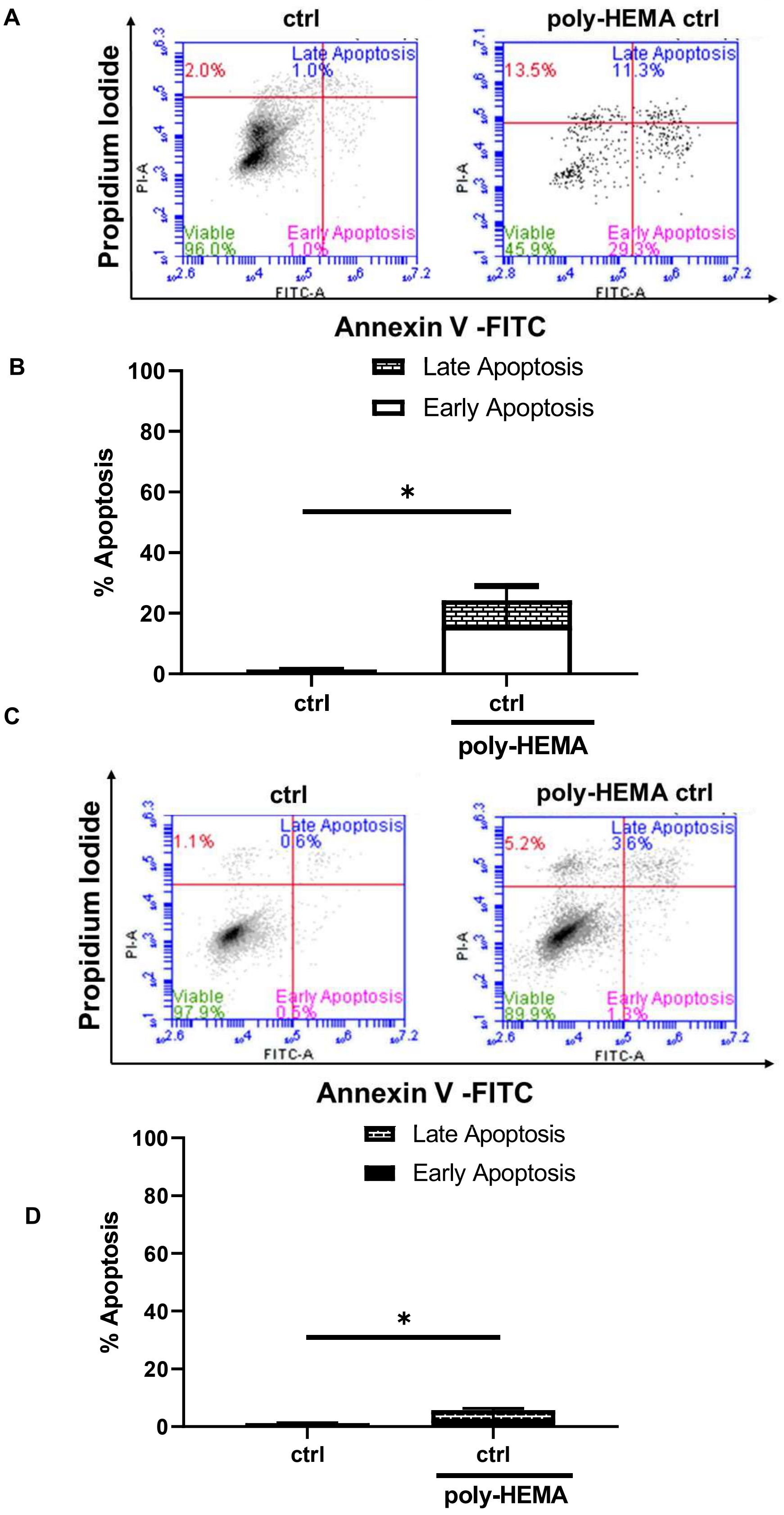

Supplement: Supplementary file 1 — Supplementary file1 (PDF 785 KB) [file 12032_2022_1664_MOESM1_ESM.pdf]
